# Charting new territory: outpatient stewardship in immunocompromised patients (first of a two-part series)

**DOI:** 10.1017/ash.2026.10780

**Published:** 2026-07-14

**Authors:** Alexander Shaffer, Rachel Bartash, Margaret E. McCort, Raneem H. Pallotta, Hannah Imlay

**Affiliations:** 1 Cleveland Clinic Foundation: Cleveland Clinic, USA; 2 Albert Einstein College of Medicine, USA; 3 University of Utah Health, USA

## Abstract

Antimicrobial stewardship (AS) among immunocompromised (IC) patients is a growing field. Since most antibiotic use occurs in the outpatient setting, antimicrobial use among IC outpatients represents an opportune area for stewardship. This two-part review explores the current state of outpatient AS in immunocompromised hosts, highlighting potential stewardship interventions and published strategies, as well as barriers in this vulnerable population. In this first section, we will highlight stewardship interventions in clinical syndromes relevant to both immunocompetent and IC hosts, including spectrum and duration of antibiotics for common bacterial infections, antibiotic allergy management, immunizations, periprocedural antimicrobial management, and multiplex polymerase chain reaction diagnostics.

## Introduction

Antimicrobial stewardship programs (ASPs) first evolved in the acute care hospital setting but have increasingly focused on other areas where antimicrobials are prescribed, including outpatient clinics and urgent care centers. Although ASPs initially focused on prescribing in the general population, stewardship principles and ASP activities are now expanding to more vulnerable populations, including immunocompromised (IC) patients, with the goal of decreasing resistant infections, reducing antimicrobial-associated side effects, and decreasing the risk of *Clostridioides difficile* infection. While successful stewardship implementation strategies include electronic medical record modifications, prescribing guidelines, provider education and feedback strategies, few interventions have focused on IC patients or syndromes. Notably, outpatient ASPs may require different infrastructure, staffing, and resources than inpatient ASPs, and much of this is unexplored among IC populations. Furthermore, well-established stewardship strategies in the general population may have less uptake in IC patients because of the perceived or real fear that these patients may have worse outcomes. However, many stewardship strategies are valuable in both populations.

In this two-part review, we discuss a nonexhaustive collection of antimicrobial stewardship (AS) opportunities and published experience for successful implementation among IC patients in the outpatient setting. We focus on patients with immunocompromise primarily due to solid organ transplant (SOT), stem cell transplant (SCT), and immunocompromising or immunomodulatory chemotherapy due to malignancy—thus, stewardship interventions may be occurring in specialty, primary care, or urgent care clinics. Part 1 focuses on common, uncomplicated infectious syndromes encountered in the setting of immunocompromise, and covers stewardship strategies for treatment (common infection syndromes, addressing allergy labels), prevention (peri-procedural antibiotics, immunizations), and diagnostics (asymptomatic bacteriuria (ASB), rapid molecular testing). Part 2 will address infectious syndromes or antibiotic utilization that occur specifically among IC patient populations.

### Antimicrobial choice and duration for uncomplicated bacterial infections

The Centers for Disease Control and Prevention Core Elements of Outpatient Antibiotic Stewardship recommend monitoring, auditing, and education focused on common infections in accordance with national clinical practice guidelines.^1^ However, IC patients are frequently excluded from clinical trials and practice guidelines that assess or make recommendations on optimal antibiotic choice or duration. As a result, the development of local guidance and audit-based stewardship interventions may be met with limited acceptance. Outpatient ASPs that include IC patients therefore need a framework for recommending antibiotic choice and duration for common infections.

Two small studies have assessed whether durations suggested for immunocompetent patients may also be safe and effective among outpatients with IC due to malignancy. Ho et al retrospectively examined optimal vs suboptimal care (defined by antibiotic choice, dose, and duration according to local guidelines) for upper respiratory tract infection (URTI), lower respiratory tract infection (LRTI), acute bacterial skin and skin structure infection (ABSSSI), and urinary tract infection (UTI) among adults at ambulatory oncology clinics and demonstrated no differences in 30 day treatment failure, delays in chemotherapy, or antibiotic associated adverse events between the groups.^2^ Importantly, they repeated their analysis to focus on duration of antibiotics only (defined as guideline-recommended durations for immunocompetent patients vs prolonged durations) and found higher rates of recurrent infection and adverse events among patients who received prolonged durations.^2^ Following this study, Arena et al examined patients with malignancy who were treated for LRTI, UTI, and ABSSSI with 5–7 days of antibiotics vs those treated for longer; short vs long duration of antibiotics was not associated with higher risk of infection-related recurrence at 30 days.^3^ There are limitations of these studies (small, single center, did not adjust for confounding by indication), but they contribute to data that suggest that institutional guidelines could target durations of antibiotics typical for immunocompetent patients for uncomplicated bacterial infections. Additionally, studies focused on more broadly IC and more acutely sick inpatients have found similar outcomes with shorter durations of therapy, suggesting that this is also a reasonable approach in IC outpatients.^4–9^

Few data evaluate whether empiric antibiotics for common infections need to be broader by default among IC outpatients without a history of MDRO, although some data exist in hospitalized patients. A recent review of microbiology in diabetic foot infections among hospitalized kidney transplant recipients (KTR) identified Pseudomonas in only 10% of cases.^10^ This prevalence is similar to that reported in the general population, suggesting empiric therapy without Pseudomonal coverage is reasonable.^10–13^ Another study found similar clinical outcomes among moderately IC hospitalized patients without prior multidrug-resistant (MDR) infection with community acquired pneumonia who received narrow spectrum therapy vs MRSA/Pseudomonas-active therapy, after adjustment in an inverse probability treatment weighting analysis.^14^ Importantly, no studies have demonstrated that empiric antibiotic choice among patients without a history of MDR organisms should differ in IC vs immunocompetent patients.^15^

Overall, these data are limited. Few studies have evaluated outpatient antimicrobial use among IC patients, and those that exist largely focus on individuals with oncologic malignancies. We could not identify any interventional study that examined the impact of stewardship strategies. Although prescribing decisions should account for patient-specific circumstances, existing data do not suggest that routine care for uncomplicated infections should substantially differ in IC vs immunocompetent patients, but more data are needed.

### Antibiotic allergy management

Antibiotic allergy labels are common, especially to beta lactams or sulfa, but on rechallenge fewer than 10% of patients may have a reproducible allergic response.^16,17^ Allergy labels directly influence antibiotic selection; IC patients with documented allergies are more likely to receive broad spectrum antibiotics, and some studies have identified worse clinical outcomes, including delays in antibiotic administration.^18–20^ One study identified a higher risk of some opportunistic infections (eg, Nocardia, Toxoplasma) in antibiotic-allergic IC patients compared to matched controls.^21^ Since antimicrobial use is common among IC patients, patients undergoing transplant evaluation or those receiving immunosuppressive agents would benefit from re-evaluation of listed allergies and potential de-labeling.

Given the limited availability of allergy specialists,^22^ allergy evaluation (by history or skin testing) and de-labeling increasingly occur among non-allergy specialists.^23^ In patients with a low-risk penicillin allergy, direct oral challenge has shown noninferiority in safety and efficacy compared to 2-stage skin testing followed by oral challenge.^24^ Antibiotic allergy decision rules including PEN-FAST and SULF-FAST have also been validated in adult IC populations.^25,26^ Several transplant centers have established organizational workflows for standardized evaluation and potential de-labeling for beta lactam and sulfa allergy labels pretransplant to improve antibiotic selection posttransplant.^19,27–29^ In a single center study by Huang et al, patients who underwent penicillin allergy evaluation consisting of oral amoxicillin challenge with or without penicillin skin testing prior to SCT were significantly more likely to receive first line antibiotics for febrile neutropenia and received antimicrobials more rapidly (66 mins vs 121 mins) than their counterparts who did not undergo allergy evaluation.^19^ Observational studies have demonstrated the effectiveness and safety of de-labeling allergies using standardized strategies among patients who are receiving immunosuppressants.^30^ If allergy evaluation cannot be completed pretransplant, posttransplant allergy de-labeling has been shown to be effective and cost-saving in some cases.^29–31^ Workflows for outpatient allergy evaluation, direct oral challenge in a nonallergist office, and potential de-labeling should be established and could be implemented into pretransplant or prechemotherapy evaluations.

### Peri-procedural antibiotics at the time of outpatient nonsurgical procedures

Although well established, collaborative guidelines for surgical prophylaxis are utilized by most centers,^32^ these guidelines are not specific to IC hosts. IC patients may have different risks for surgical site infections due to their impairments in immune function, prior exposure to antibiotics and frequent colonization with MDR organisms. Given these risk factors, IC patients may be perceived as needing longer and/or broader courses of perioperative antibiotics, though there is limited data to support this, and no well-established guidelines with such recommendations.^32,33^

The role of nonoperative periprocedural antibiotics in IC patients is even less clear, despite the frequency of these outpatient procedures. The most well-established guidelines are for dental prophylaxis, published by the American Heart Association, which recommend antibiotics for patients most likely to develop endocarditis in the setting of structural cardiac abnormalities, and immunocompromising conditions in and of themselves are not considered to be a risk factor.^34^

Despite clear guidelines, a recent study evaluating appropriateness of dental prophylaxis within the Veterans Affairs (VA) system found that the majority of prescriptions were inappropriate, including 4% of prescriptions written for “immunosuppressive conditions.”^35^ We suspect overuse is not unique to the VA system, particularly in the community where providers may have less experience caring for IC patients and therefore apprehensive regarding infectious risks. Even in the authors’ experiences, prophylaxis for dental procedures is not uncommon among IC patients without other risk factors at their institutions, suggesting prescribed prophylaxis often falls outside of the established guidelines. This practice may be an opportunity for ASPs to review prescribing patterns at their institutions, provide prescriber education, and update protocols highlighting appropriate use.

Other commonly performed outpatient procedures in SOT recipients include kidney, liver and cardiac biopsies to monitor for rejection. Despite the frequency of these procedures, there are no established guidelines recommending for or against the use of prophylactic antibiotics. The National Kidney Foundation curriculum does not mention periprocedural antibiotics for native kidney biopsy,^36^ and evidence suggests that this can safely be performed without antibiotics.^37^ Despite this, up to 20% of centers administer prophylactic antibiotics in native kidney biopsies.^38^ This discrepancy may be larger among KTR, though we know of no published data. Another commonly performed outpatient procedure for KTR is ureteral stent removal, which occurs a few weeks posttransplant. While guidelines suggest prophylaxis be considered for similar procedures, particularly when additional risk factors for infection are present,^39^ some studies evaluating the benefit of antimicrobial prophylaxis in the setting of kidney transplantation suggest no benefit to additional antibiotics, other than standard SMX-TMP for pneumocystis.^40,41^ The use of prophylaxis for ureteral stent removal may be an area for ASP interventions, such as provider education and protocol development.

Data regarding antibiotic prophylaxis for liver biopsy in liver transplant recipients are scarce. Two retrospective reviews of liver biopsy outcomes in transplant recipients found that while the overall periprocedural infectious risk was low, infection occurred more frequently in patients who underwent choledochojejunostomy during transplant.^42,43^ Given these findings, guidelines from the UK recommend against the use of routine antibiotics for liver biopsy, except in the presence of biliary-enteric anastomoses or previous infectious complications from liver biopsy,^44^ though the American Association for the Study of Liver Diseases does not recommend for or against the use of periprocedural antibiotics.^45^

Future studies should evaluate the necessity of periprocedural antibiotics for routine procedures in immunocompromised (IC) patients. With current data, ASPs should evaluate their own guideline-concordant prescribing and administer provider feedback as there is potential for variable practice and overuse in this setting.

### Immunizations as stewardship interventions among immunocompromised patients

As a stewardship strategy, immunizations have been postulated to impact antimicrobial resistance (AMR) directly and indirectly by reducing antibiotic use. Vaccination can directly impact AMR by reducing circulation of infections caused by the vaccine target.^46^ Pneumococcal vaccination is an example of the direct effects of vaccination on AMR—studies of invasive pneumococcal disease, primarily in children, have demonstrated both fewer antibiotic prescriptions^47–49^ and fewer reduced-susceptibility infections^46,50^ following introduction of pneumococcal vaccines or vaccine campaigns, although some studies of adults ≥65 do not demonstrate the same impact.^51,52^

Vaccines may also impact AMR indirectly—by either reducing secondary infections or by reducing febrile illnesses that often lead to the use of antibiotics.^46^ This indirect impact on antimicrobial prescribing and AMR has been best studied with influenza vaccination. A meta-analysis by Buckley et al demonstrated that influenza vaccination reduced antibiotic prescribing for acute respiratory infections for influenza-vaccinated vs unvaccinated populations;^53^ this association has also been noted in observational studies.^46,54–56^

Studies evaluating the efficacy of certain vaccines in IC patients have demonstrated reduced risk or severity of their targeted disease, although IC patients may require different vaccine strategies (eg, higher or multiple doses).^57–61^ We found no studies that evaluated the direct or indirect impact of vaccination on antibiotic use or AMR among an IC population; however, it is plausible that if appropriately dosed vaccination is effective, it may also reduce antibiotic use and impact AMR. Overall, establishing center practice guidelines that encourage the use of vaccines may improve rates/severity of infections and plausibly decrease unnecessary antimicrobials.

### Diagnostic stewardship and asymptomatic bacteriuria after kidney transplant

Well-established guidelines recommend against routine urine culture in asymptomatic KTR,^62,63^ as studies have shown a lack of benefit of treating ASB or candiduria.^64–67^ Based on these data and guideline recommendations, many transplant centers have moved away from surveillance cultures.

However, many institutions have implemented hospitalwide reflex urine culture as a diagnostic stewardship intervention. This practice restricts urine cultures unless certain criteria are met on urinalysis, including the presence of white blood cells and bacteria, with the goal of decreasing antibiotic use in ASB. While this may reduce the overall numbers of urine cultures performed on an institutional level,^68,69^ less is known about the impact on KTRs specifically. In this population, urinalyses are often abnormal for reasons other than infection, including graft rejection and/or recurrent glomerular disease. Given this, along with the high frequency of ASB, it can be challenging to interpret the clinical significance of “positive” UAs reflexed to culture in KTRs and may lead to increased unnecessary antibiotic use for ASB.

A single-center study of 709 urinalyses in KTRs ≤3 months posttransplant found reflexed cultures in 35% of urine samples.^70^ Of these, 40 cultures were positive, with 34 classified as ASB.^70^ Despite this classification, almost 15% of the patients with ASB received treatment with antibiotics.^70^ The authors concluded that the majority of ASB diagnosed posttransplant was a result of reflexed urine culture, suggesting a potential stewardship target.^70^

While these findings are limited to a single center, they may reflect a wider stewardship challenge, given the number of institutions that perform reflexed urine cultures. We suggest that even though using a UA with reflex to urine culture may reduce the number of urine cultures performed, transplant centers should reconsider utilizing reflex cultures among KTRs given the high likelihood of abnormal urinalyses leading to potentially unnecessary treatment of ASB. Rather, centers should only order urine cultures if patients have UTI symptoms. Should routine monitoring of urinalyses be indicated from a transplant perspective to evaluate for underlying renal pathology, stewardship programs could remove reflex cultures from these orders to prevent diagnostic uncertainty of positive cultures in the absence of symptoms with the goal of decreasing unnecessary antibiotic use.

### Diagnostic stewardship of respiratory and GI multiplex panels among immunocompromised patients

Respiratory viral infections (RVIs) are a common cause of morbidity in IC hosts. While data on outpatient incidence are limited, a longitudinal cohort study in Switzerland found that 34% of RVIs in SOT recipients required admission, suggesting the majority are diagnosed and managed as outpatients.^71^ Outpatient management is enabled by the use of naso/oropharyngeal multiplex polymerase chain reaction (mPCR) platforms, which can rapidly diagnose RVIs^72^ and allow for appropriate initiation of antiviral therapy.^73^ Rapid diagnosis of SARS-CoV2 and influenza is particularly important for IC hosts who are at risk for severe disease, as early treatment with antivirals leads to improved outcomes.^74–76^ However, the clinical value of broader multiple mPCR-based assays is unclear, as many detect respiratory viral pathogens for which no treatment exists. ^77^

Whether mPCR testing for RVIs can improve ASP remains unclear—potentially, the identification of a virus as a cause of illness may dissuade inappropriate antibiotic use. Inappropriate antibiotic prescribing for respiratory infections is common in the outpatient setting, with studies suggesting that close to half of prescriptions are inappropriate.^78^ While numerous studies have focused on ASP interventions in this setting with improved prescribing seen with patient education, electronic decision support and other stewardship tools, these studies have generally focused on immunocompetent hosts.^79^ Multiplex PCR panels could be a potential stewardship tool if a viral pathogen is identified; however, recent studies, including randomized controlled trials (RCTs), have generally failed to show a decrease in antibiotic prescribing with their use.^73,80–83^ Notably, a single center RCT found an association of mPCR testing with faster implementation of pathogen-directed care,^80^ and a prospective cohort study conducted at a high-volume urgent care center showed a 15-pathogen respiratory panel with integrated ASP interventions led to a lower rate of antibiotic prescribing in patients presenting with URTI compared to a seasonally matched historical cohort.^84^ While only a handful of patients were on immunosuppressants, these findings suggest that diagnostic tools integrated with ASP interventions may improve stewardship outcomes.

Data on mPCR testing specifically in IC hosts is limited. A cohort study by Krantz et al evaluated viral testing in ambulatory oncology patients and found that, while one-third of patients received antibiotics when presenting with URI symptoms, the likelihood of antibiotic prescribing was reduced if viral testing was performed on the day of presentation, though practice varied significantly by provider.^85^ However, Mercuro et al reported that, in the inpatient setting, antibiotics were only discontinued in 14% of positive cases following rapid viral testing,^86^ suggesting that in IC hosts, concern for superimposed bacterial pneumonia may limit potential stewardship benefits of RVI testing.

Given limited treatment options for the majority of RVIs, the high cost of tests, and limited data to support improved antibiotic prescribing, many institutions have implemented diagnostic stewardship measures limiting access to broader mPCR platforms in patients with suspected RVIs.^72^ Targeted mPCR testing for treatable pathogens, including influenza and SARS-CoV2, is likely not only more clinically impactful and cost-effective, but also more accessible, as these tests are easier to implement in the outpatient setting. Other potential targeted stewardship interventions for improving outpatient prescribing include patient education on indications for antibiotic therapy and peer comparison dashboards, which could be implemented alongside mPCR stewardship in the management of IC hosts. While it should be noted that there may be other reasons for RVI testing, including infection prevention practices and isolation policies, these are mostly applicable to the inpatient setting and therefore, outpatient stewardship of mPCR testing should not impact these practices.

Multiplex PCR tests are also widely available for stool specimens in patients with suspected acute gastrointestinal (GI) infections. However, a limitation of mPCR diagnostics is their inability to distinguish active infection from previous infection, which is of particular concern in IC hosts where prolonged viral shedding is common.^87^ Further, noninfectious causes of their GI symptoms, including medication side effects, are common. Spurious identification of organisms in these cases may instead lead to an increase in antibiotic prescribing.^88^ In a small inpatient study of liver transplant recipients, 71% of positive PCR and 29% of negative PCR results led to a change in antibiotic therapy (escalation or de-escalation).^89^ Given the limitations of discerning active infection, potential for increase in antibiotic use and high costs of these tests, GI mPCR panels are an optimal diagnostic stewardship target.^88^ Institutions may consider delaying testing among stable outpatients and monitoring for resolution of symptoms with conservative management, especially as many infectious causes of diarrhea are self-limited, even in IC hosts.

## Conclusions

Though more data are needed to guide optimal stewardship practices among IC outpatients, based on the available literature and the deleterious effects of inappropriate antibiotic use, IC patients should not be excluded from ASP practices. Other important stewardship opportunities not covered in this review include review of antibiotic choice and duration at discharge; review of cultures obtained in the ambulatory setting; role of primary care physicians in antibiotic use, especially in resource limited outpatient settings; and maintenance of an active outpatient antimicrobial therapy program with consideration of PO transitions. In part 2 of our review, we will delve further into opportunities for stewardship in unique syndromes in IC patients.

## Supporting information

10.1017/ash.2026.10780.sm001 Shaffer et al. supplementary material Shaffer et al. supplementary material

## Supplementary material

The supplementary material for this article can be found at https://doi.org/10.1017/ash.2026.10780.
