# Supplementary material for: Charting new territory: outpatient stewardship in immunocompromised patients (first of a two-part series)
Source: Antimicrob Steward Healthc Epidemiol. 2026 Jul 14;6(1):e213. doi: 10.1017/ash.2026.10780 (PMC13372734; doi:10.1017/ash.2026.10780)
Supplement: Shaffer et al. supplementary material [file S2732494X26107803sup001.docx]

**Outpatient Stewardship in Immunocompromised Hosts: Part 1**

Antimicrobial choice and duration for uncomplicated bacterial infections

- Longer duration of antibiotics for LRTI, UTI and ABSSSI in patients with malignancies may be associated with higher rates of recurrent infections and adverse events compared to shorter regimens.
- Empiric antibiotics for IC patients with common bacterial infections (such as DFI or CAP) need not be broader than general population with same infection.

Antibiotic allergy management

- Patients undergoing transplant evaluation and those receiving immunosuppressive agents likely benefit from allergy de-labeling where appropriate.
- Validated antibiotic allergy assessment tools and oral challenges can be used in IC outpatients as they are used in general population.
- Allergy de-labeling prior to transplantation may lead to faster initiation of first line empiric antibiotics and cost savings after transplant.

Peri-procedural antibiotics the time of outpatient procedures

- Expert guidelines regarding antibiotic prophylaxis for non-surgical outpatient procedures (such as AHA dental prophylaxis guidelines, native kidney biopsy, ureteral stent removal, and liver biopsy) do not include immunocompromise as an indication for antibiotics.
- Despite guideline recommendations, antibiotic use for non-surgical outpatient procedures in IC patients is inappropriately high.

Immunizations as stewardship interventions among immunocompromised patients

- Immunizations have been shown to impact AMR directly, especially in the case of pneumococcal vaccination in children, by reducing antibiotic use.
- Influenza vaccines have been shown to indirectly reduce antimicrobial prescribing among vaccinated vs unvaccinated populations.
- While we found no studies demonstrating indirect or direct impact of vaccination on antimicrobial use or AMR among IC patients, it is plausible that vaccines in IC patients also lead to reduced antibiotic use.

Diagnostic stewardship and asymptomatic bacteriuria after kidney transplant

- Based on data showing lack of benefit to treatment of asymptomatic bacteriuria in KTR, many centers no longer check surveillance urine cultures in this group.
- However, the introduction of reflex urine cultures (in response to abnormal urinalysis) at the institutional level may be leading to a new increase in asymptomatic bacteriuria identification and treatment in KTR, and should be avoided.

Diagnostic stewardship of respiratory and GI multiplex panels among immunocompromised patients

- Among immunocompetent population, multiplex PCR panels to diagnose respiratory viral infections may not reduce inappropriate antibiotic prescriptions unless combined with provider education or decision support tools.
- There is less data on the use of mPCR viral testing in IC or outpatients, but a study in oncology patients found that antibiotics were often prescribed despite detection of a viral pathogen
- Similarly, stool mPCR testing may not positively impact antimicrobial stewardship, as it may lead to spurious identification of organisms when the diarrhea had an alternative, non-infectious cause.
- Limiting mPCR testing in the outpatient IC population is recommended.
